# Supplementary material for: High thermoelectric performance enabled by convergence of nested conduction bands in Pb7Bi4Se13 with low thermal conductivity
Source: Nat Commun. 2021 Aug 9;12:4793. doi: 10.1038/s41467-021-25119-z (PMC8352968; doi:10.1038/s41467-021-25119-z)
Supplement: Supplementary file 1 — Supplementary Information [file 41467_2021_25119_MOESM1_ESM.pdf]

Supplementary Information

High thermoelectric performance enabled by convergence of nested conduction bands in $Pb_7Bi_4Se_{13}$ with low thermal conductivity

Lei Hu,[1,2]* Yue-Wen Fang,[2] Feiyu Qin,[2] Xun Cao,[1] Xiaoxu Zhao,[1] Yubo Luo,[1] Durga Venkata Maheswar Repaka,[3] Wenbo Luo,[4] Ady Suwardi,[3] Thomas Soldi,[5] Umut Aydemir,[6,7] Yizhong Huang,[1] Zheng Liu,[1] Kedar Hippalgaonkar,[1,3] G. Jeffrey Snyder,[5] Jianwei Xu,[3] Qingyu Yan[1]*

1 School of Materials Science and Engineering, Nanyang Technological University, Singapore 639798, Singapore

2 Materials and Structures Laboratory, Tokyo Institute of Technology, Yokohama, 226-8503, Japan

*3 Institute of Materials Research and Engineering, A*STAR (Agency for Science, Technology and Research), 138634, Singapore*

4 Institute for Advanced Materials, North China Electric Power University, Beijing 102206, China

5 Department of Materials and Science Engineering, Northwestern University, Evanston, IL 60208, United States

6 Department of Chemistry, Koc University, Sariyer, Istanbul 34450, Turkey

7 Koc University Boron and Advanced Materials Application and Research Center, Sariyer, Istanbul 34450, Turkey

Corresponding author: leihuedu@gmail.com AlexYan@ntu.edu.sg

Supplementary Note 1: Thermoelectric measurements and thermomechanical investigations

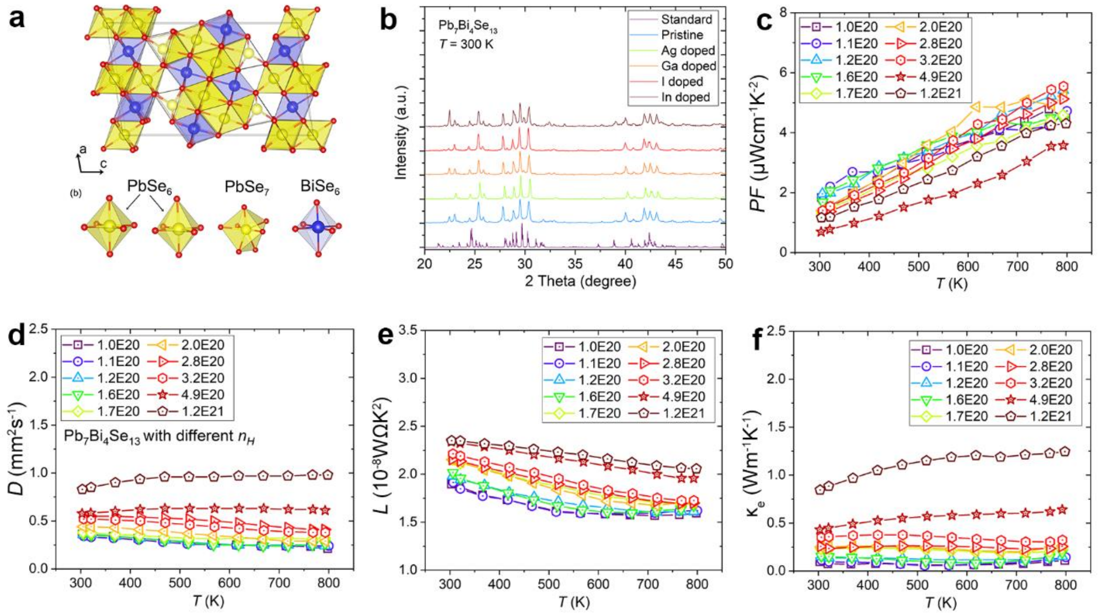

Fig. S1. **a** Crystal structure of $Pb_7Bi_4Se_{13}$, which consists of diverse polyhedra, $PbSe_6$, $PbSe_7$ and $BiSe_6$. **b** XRD patterns of standard, pristine, Ag-, Ga-, I- and In-doped $Pb_7Bi_4Se_{13}$. **c** Power factor (*PF*), **d** thermal diffusivity, *D*, **e** Lorenz number, *L*, and **f** electronic thermal conductivity.

Table S1. Sample composition, carrier concentration, charge mobility and density

Composition	Notation	n_H (cm^{-3})	u_H (cm^{2}V^{-1}s^{-1})	Density (gcm^{-3})
$Pb_7Bi_4Se_{13}$	Pristine	3.2E20	10.2	7.77
$(Pb_{0.98}Ga_{0.02})_7Bi_4Se_{13}$	Ga1	2.0E20	11.8	7.61
$(Pb_{0.95}Ga_{0.05})_7Bi_4Se_{13}$	Ga2	1.2E20	12.8	7.51
$(Pb_{0.9}Ga_{0.1})_7Bi_4Se_{13}$	Ga3	1.0E20	10.3	7.50
$(Pb_{0.95}In_{0.05})_7Bi_4Se_{13}$	In1	2.8E20	8.8	7.56
$(Pb_{0.9}In_{0.1})_7Bi_4Se_{13}$	In2	4.9E20	7.8	7.59
$(Pb_{0.95}Ag_{0.05})_7Bi_4Se_{13}$	Ag1	1.6E20	9.7	7.72
$(Pb_{0.9}Ag_{0.1})_7Bi_4Se_{13}$	Ag2	1.1E20	11.4	7.57
$Pb_7Bi_4Se_{12.6}I_{0.4}$	I1	1.7E20	12.9	7.57
$Pb_7Bi_4Se_{12.2}I_{0.8}$	I2	1.2E21	6.3	7.56

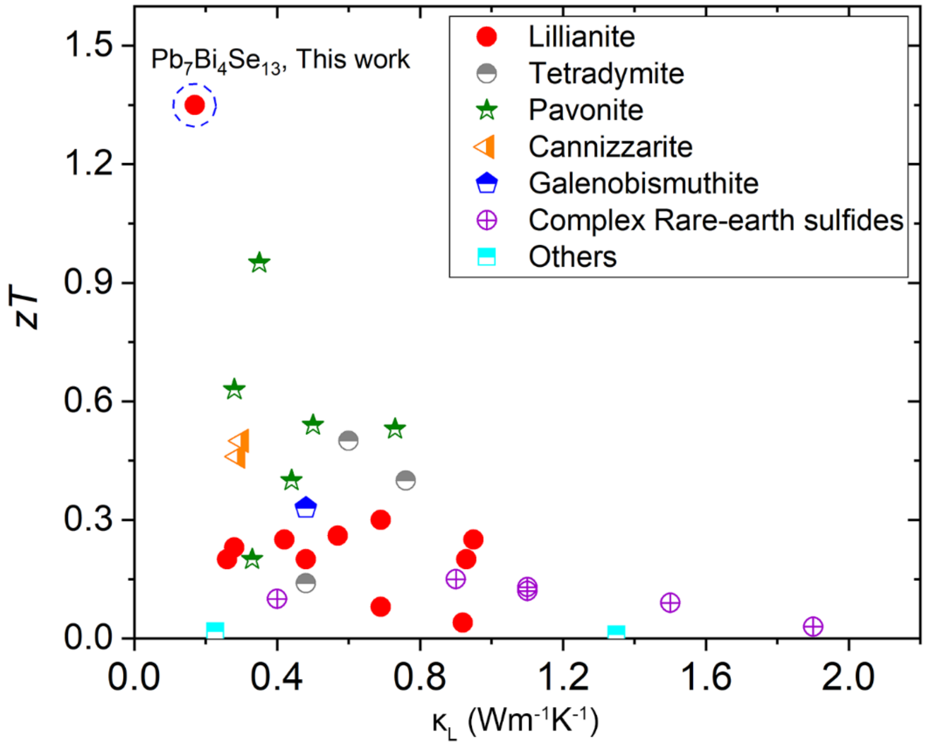

Fig.S2. Lattice thermal conductivity, κ_L and zT values and of lillianite, tetradymite, pavonite, cannizzarite, galenobismuthite, complex rare-earth sulfides and other structure-similar compounds.

After searching for compounds with similar lillianite-type structures, 37 different compounds (including $Pb_7Bi_4Se_{13}$ here) are found and compared. As shown in Fig. S2, lattice thermal conductivity, κ_L and zT values and of lillianite (red circle), tetradymite (half gray circle), pavonite (half green star), cannizzarite (half triangle), galenobismuthite (half pentagon), complex rare-earth sulfides (cross circle) and structure-similar other compounds (half cyan square) are presented. It is obvious that the $Pb_7Bi_4Se_{13}$ marked by the blue circle not only demonstrates the highest zT value among lillianite type structures, but also exhibits the competitive thermoelectric performance, compared to other compounds with similar structures. The detailed parameters of these compounds shown in Fig. S2 are tabulated in Table S2.

TableS2. Lattice thermal conductivities and *zT* values of compounds with lillianite-similar structures

Compound	κ_L ($Wm^{-1}K^{-1}$)	*zT*	*T* (K)	Category	Literature
$Pb_3Bi_2S_6$	0.57	0.26	715		1
$Ag_2Pb_6Bi_{10}Se_{22}$	0.28	0.23	523		2
$Pb_6Bi_2Se_9$	0.95	0.25	673		3
$SnPb_2Bi_2S_6$	0.69	0.3	770		4
$Pb_5Bi_{12}Se_{23}$	0.42	0.25	723		5
$Pb_5Bi_{18}Se_{32}$	0.48	0.2	723		5
$Sn_4Bi_{10}Se_{19}$	0.26	0.2	535		6
$Sn_4Bi_2Se_7$	0.92	0.04	450	Lillianite	7
$Sn_2Bi_2Se_5$	0.69	0.08	525		7
$SnBi_4Se_7$	0.93	0.2	673		7
ǂ$Sn_2Pb_5Bi_4Se_{13}$	--	--	300		8
ǂ$Sn_{8.65}Pb_{0.35}Bi_4Se_{15}$	--	--	300		8
‖$K_xSn_{62x}Bi_{2+x}Se_9$	--	--	--		9
‖$KSn_5Bi_5Se_{13}$	--	--	--		9
§$LiPbSb_3S_6$	0.21	--	723		10
$PbBi_2Te_4$	0.76	0.4	650		11
$PbBi_4Te_7$	0.6	0.5	600	Tetradymite	11
$PbSb_2Te_4$	0.48	0.14	340		12
$CdSnBi_4Se_8$	0.44	0.4	850		13
$CdPbBi_4Se_8$	0.28	0.63	850		13
$Cu_{1.61}Bi_{4.81}S_8$	0.33	0.2	675		14
$CdPb_2Bi_4S_9$	0.73	0.53	775	Pavonite	15
$CdAg_2Bi_6Se_{11}$	0.35	0.95	775		15
$LiSn_2Bi_5S_{10}$	0.5	0.54	825		16
*$NaSn_2Bi_5S_{10}$	--	--	--		16
$Pb_5Bi_6Se_{14}$	0.29	0.46	705	Cannizzarite	17
$Pb_5Bi_6Se_{14-x}I_x$	0.3	0.5	723		17
$PbBi_2S_4$	0.48	0.33	710	Galenobismuthite	1
$(GdS)_{1.20}NbS_2$	1.5	0.09	873		18
$(Gd_{0.5}Dy_{0.5}S)_{1.21}NbS_2$	1.1	0.13	873		18
$(DyS)_{1.22}NbS_2$	1.1	0.12	873	Complex rare-earth sulfides	18
$(GdS)_{0.60}NbS_2$	1.9	0.03	873		18
$(LaS)_{1.14}NbS_2$	0.9	0.15	950		18
$(Yb_2S_2)_{0.62}NbS_2$	0.4	0.1	300		18
$MnSb_2Se_4$	1.35	0.01	300	Others	19
$MnPb_{16}Sb_{14}S_{38}$	0.227	0.018	725		20
**$Pb_7Bi_4Se_{13}$**	**0.17**	**1.35**	**800**	**Lillianite**	**This work**

ǂ For $Sn_2Pb_5Bi_4Se_{13}$ and $Sn_{8.65}Pb_{0.35}Bi_4Se_{15}$, only electrical properties were measured and reported. In details, their electrical conductivities were measured from around 25 K to 300 K, while their

Seebeck coefficients were collected from around 300 K to 675 K. No thermal conductivities are available. Here only the room temperature electrical conductivities, about 14 Scm^{-1} and 10 Scm^{-1} for $Sn_2Pb_5Bi_4Se_{13}$ and $Sn_{8.65}Pb_{0.35}Bi_4Se_{15}$ are extracted. Also, -200 μVK^{-1} and 130 μVK^{-1} for $Sn_2Pb_5Bi_4Se_{13}$ and $Sn_{8.65}Pb_{0.35}Bi_4Se_{15}$ are obtained.

‖ For $K_xSn_{62x}Bi_{2+x}Se_9$ and $KSn_5Bi_5Se_{13}$, the crystal structure data was report without the measurement of electrical and thermal properties.

§ For $LiPbSb_3S_6$, only crystal structure and thermal conductivity are available.

* For $NaSn_2Bi_5S_{10}$, its band gap is 0.07 eV, whose electrical and thermal properties are not measured.

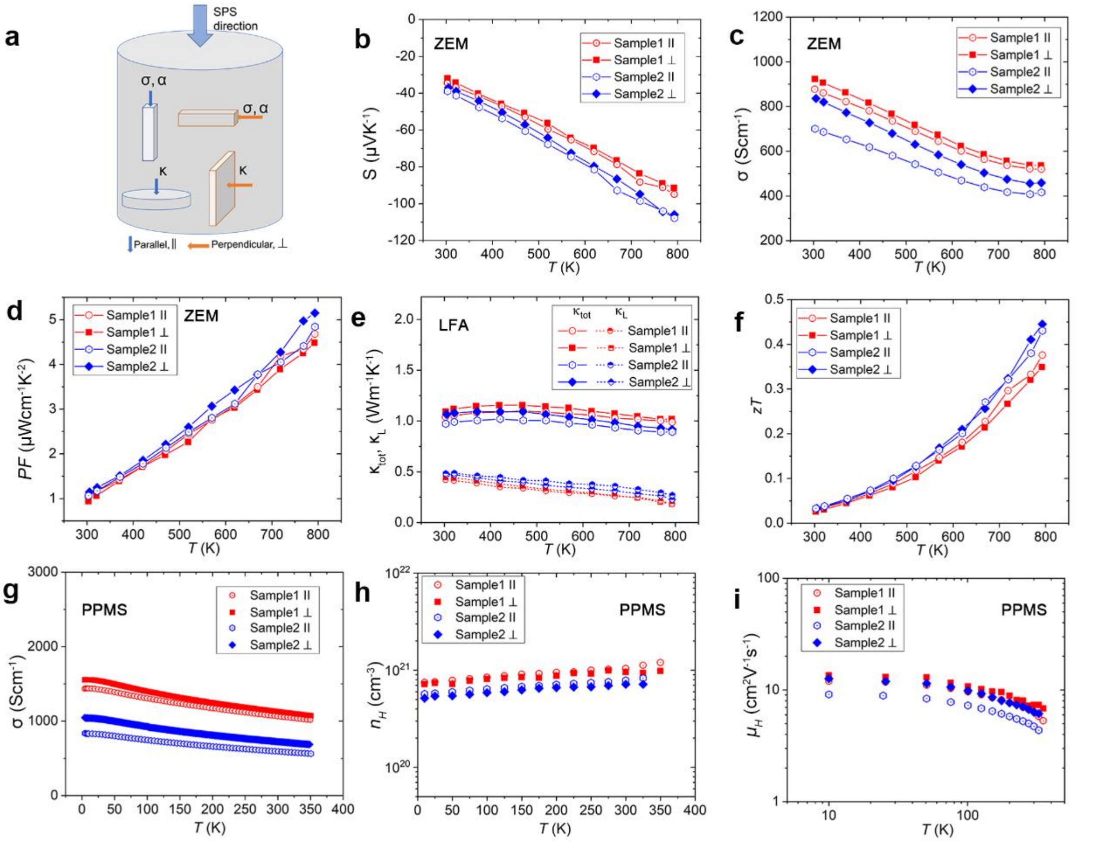

Fig. S3. Thermoelectric properties of $Pb_7Bi_4Se_{13}$ with $n_H = 9.2\times10^{20}$ cm^{-3} (Sample 1) and 7.4×10^{20} cm^{-3} (Sample2) parallel (∥) and perpendicular (⊥) to the SPS direction. **a** Measurement geometry, **b** Seebeck coefficient, **c** electrical conductivity, **d** power factor (*PF*), **e** total and lattice thermal conductivity, **f** *zT* values, **g** electrical conductivity at low temperatures, **h** Hall carrier concentration, **i** mobility. The thermoelectric properties were measured on ZEM device and the Hall measurements were completed on PPMS instrument.

As shown in Fig. S3, the measurement geometry shows the electrical and thermal conductivities measured parallel and perpendicular to the SPS direction. The Seebeck coefficient, electrical conductivities, thermal conductivity and *zT* values of two different compositions were measured from RT to 800 K, as shown in Fig. S3b - 3f. Electrical conductivities, Hall carrier concentration and mobility were also measured and presented in Fig. S3g-3i. It is clear that there exists a small degree of anisotropy of thermoelectric properties, due to the non-cubic crystal structure of $Pb_7Bi_4Se_{13}$. Meanwhile, the *PF* and thermal conductivities in two different measurement directions are close, as shown in Fig. S3d-3e, which leads to the similar *zT* values in the two directions.

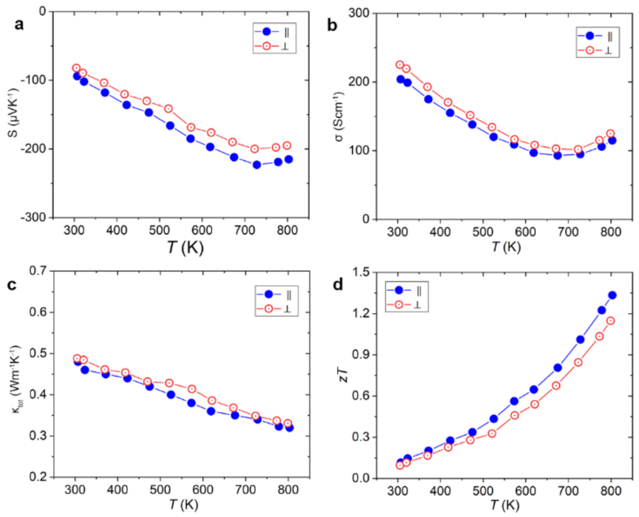

Fig.S4. Thermoelectric properties of $Pb_7Bi_4Se_{13}$ with $n_H = 1.2\times10^{20}$ parallel (∥) and perpendicular (⊥) to the SPS direction. **a** Seebeck coefficient, **b** electrical conductivity, **c** total thermal conductivity, **d** *zT* values.

To clarify the anisotropy of thermoelectric properties of $Pb_7Bi_4Se_{13}$ with $n_H = 1.2\times10^{20}$ cm^{-3}, we present the Seebeck coefficient, electrical conductivities, thermal conductivities and *zT* values measured parallel and perpendicular to the SPS direction. The measurement geometry is presented in Fig. S3a. Clearly, there exist a certain degree of anisotropy of thermoelectric properties as shown in Fig. S4a-4c. As shown in Fig. S4d, the thermoelectric performance parallel to the SPS direction is slightly better than that perpendicular to the SPS direction. For convenience of discussion, thermoelectric properties of doped $Pb_7Bi_4Se_{13}$ in the main text are results parallel to the SPS direction.

In this paragraph, a detailed discussion on the anisotropy of thermoelectric properties is performed. Thermoelectric materials with non-cubic structures demonstrate different degrees of anisotropy of charge and phonon transport. The anisotropy is considerably large in some layered structures, such as Bi_2Se_3, BiSe and their derivates.[21, 22, 23] $Pb_5Bi_6Se_{14}$, a member of cannizzarite-type structure, also exhibits different electrical and thermal conductivities in parallel and perpendicular directions.[17] It mainly originates from its structural features, in which PbSe and Bi_2Se_3 subunit stack alternatively along the *c* axis. This layered structure is easily to form preferred orientation in the pressing direction. In contrast, other compounds, such as $Pb_3Bi_2S_6$,[1] $SnPb_2Bi_2S_6$ (members of lillianite homologous series),[4] and $PbBi_2S_4$ (a member of

galenobismuthite homologous series),[1] demonstrate nearly isotropic electrical and thermal transport properties. Taking $Pb_3Bi_2S_6$ as an instance, it consists of NaCl-type (Pb/Bi)S layers with a mirror as twinning operation. These layers form the NaCl-type strips and avoid the formation of single layered structure preferred stacking to any crystal axis. Under pressing, crystal grains tend to distribute randomly, which gives rise to the near isotropy of electrical and thermal properties of parallel and perpendicular directions. For $Pb_7Bi_4Se_{13}$, its crystal structure includes the NaCl-type (Pb/Bi)Se strips without separate PbSe and BiSe layer stacking alternatively. This might give rise to this anisotropy of thermoelectric properties. In total, the anisotropy of electrical and thermal transport properties is structural dependence. Nearly isotropic electrical and thermal transport properties are also available in $Pb_3Bi_2S_6$, $SnPb_2Bi_2S_6$, and $PbBi_2S_4$. $Pb_7Bi_4Se_{13}$ indeed demonstrates a certain degree of anisotropy in electrical and thermal conductivities.

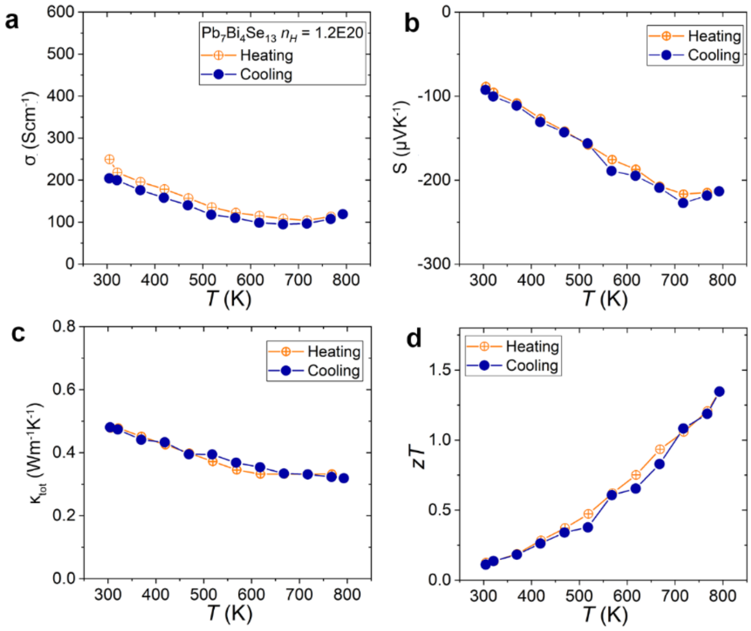

Fig. S5. **a** Electrical conductivity, **b** Seebeck coefficient, **c** thermal conductivity, **d** zT of $Pb_7Bi_4Se_{13}$ with $n_H = 1.2\times10^{20}$ cm^{-3} (Ga2) from heating and cooling measurements.

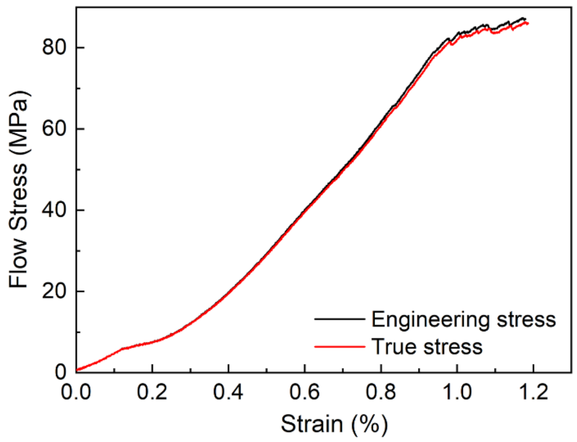

Fig. S6. The compressive strain–stress curves of $(Pb_{0.95}Ga_{0.05})_7Bi_4Se_{13}$. The strain rate is 5×10^{-4} s^{-1}.

The result of compression test in Fig. S6 indicates the strength and strain of $(Pb_{0.95}Ga_{0.05})_7Bi_4Se_{13}$. The UCS (ultimate compressive strength) of the $(Pb_{0.95}Ga_{0.05})_7Bi_4Se_{13}$ is about 85 MPa and the strain at fracture is about 1.2%.

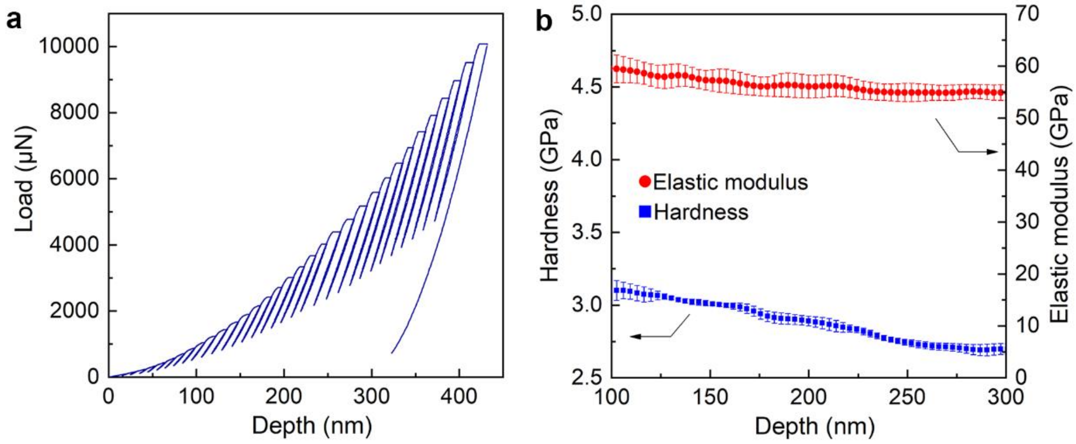

Fig. S7. Nanoindentation curves of $(Pb_{0.95}Ga_{0.05})_7Bi_4Se_{13}$. **a** Load/unload-depth curve, 33 cycles for one point. **b** Hardness and elastic modulus at different depths. The standard error originates from averaging data from 5 different samples with the same composition.

The nanoindentation load/unload-depth curve is presented in Fig. S7, which includes 33 cycles for one point. For the final result, 10 points for one sample and 5 samples have been measured and averaged. The hardness and elastic modulus at different depths are exhibited in Fig. S7b. The hardness is estimated to be 2.88±0.04 GPa, and the elastic modulus is 56±4 GPa. Both the hardness and modulus reduce slightly with increasing the depth, due to size effect.

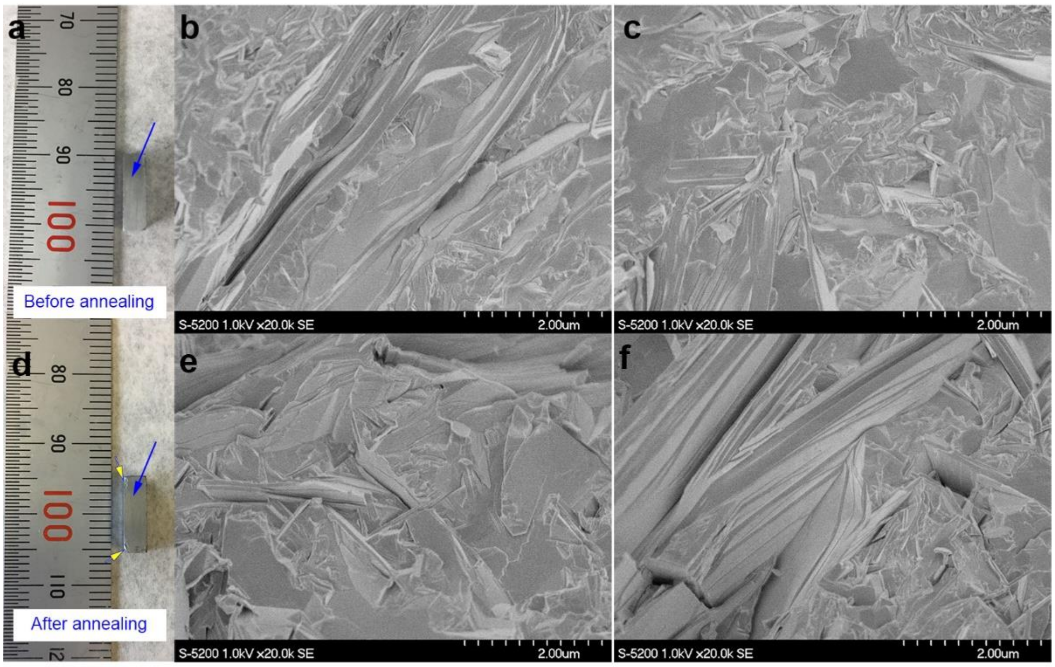

Fig. S8. Microstructural investigation of thermal annealing of $(Pb_{0.95}Ga_{0.05})_7Bi_4Se_{13}$. **a** Photograph, **b** and **c** SEM images of ingot before annealing. **d** Photograph, **e** and **f** SEM images of ingot after annealing on 800 K in vacuum condition for 336 hours (2 weeks). The blue arrows indicate the ingot surface. The small yellow arrows mark the tiny cracks in the corners after thermal annealing.

To investigate the thermal stability of $(Pb_{0.95}Ga_{0.05})_7Bi_4Se_{13}$, this ingot was subjected to the annealing at 800 K for 336 hours (2 weeks) in vacuum quartz tube. The microstructural features of the ingot before and after thermal annealing are compared. Compared to the ingot before annealing as shown in Fig. S8a, the surface of the ingot (indicated by the blue arrow) after annealing exhibits the features of completeness and smoothness, without pitting and bloating as revealed in Fig. S8d. Only small cracks are observed in the ingot corners marked by the small yellow arrows. SEM images shows fracture surfaces before (Fig. S8b-c) and after annealing (Fig. S8e-f). It is obvious that the fracture surfaces after annealing share similar features with its counterpart, in which no obvious micropores can be detected. These investigation shows the $(Pb_{0.95}Ga_{0.05})_7Bi_4Se_{13}$ exhibits reasonably robust thermal stability.

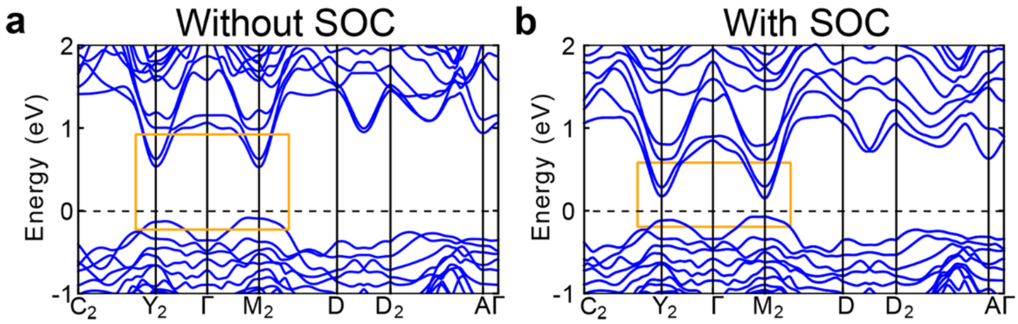

Fig. S9. Electronic band structures of $Pb_7Bi_4Se_{13}$ calculated by **a** standard DFT without SOC and **b** DFT with SOC. Fermi level is set to zero.

Fig. S9 shows band structures calculated by DFT without SOC (*i.e.*, Fig. 2a in the main text) and with SOC. Although the SOC is found to suppress the band gap from 0.67 eV (without SOC) to 0.22 eV (with SOC), it has discernable effect on the band dispersions near the conduction band minima (CBM) and valence band maxima (VBM) as highlighted by the orange squares, indicating that the low-energy electronic states do not be affected significantly with the inclusion of SOC.

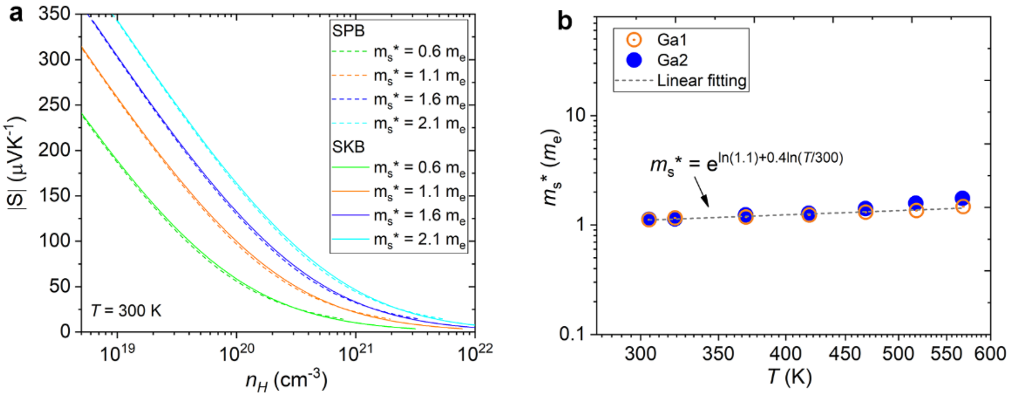

Fig. S10. **a** Hall carrier concentration as a function of Seebeck coefficient (Pisarenko plot) by using single parabolic band (SPB) and single Kane band (SKB) models with different effective mass. **b** Temperature dependence of effective mass of two compositions, Ga1 and Ga2.

By using a series of effective mass, m_s^*, Hall carrier concentration as a function of Seebeck coefficient (Pisarenko plot) at 300 K is presented in Fig. S10a. The SPB and SKB models are established based on the assumption of the dominant acoustic phonon scattering (APS). For the prediction of Seebeck coefficient, both SPB and SKB could equally work well with the same effective mass, also observed in $PbTe_{1-x}I_x$.[24] Actually, the m_s^* of $Pb_7Bi_4Se_{13}$ compounds are increasing with increasing temperature, consistent with the Kane band feature. This temperature dependence of m_s^* is also reported in PbTe thermoelectric materials with Kane band feature,[25] in which the SKB model is utilized to describe its Seebeck coefficient and mobility.

Supplementary Note 2: Quality factor analysis based on single parabolic band

Quality factor based on single parabolic band assumption and acoustic phonon scattering dominating charge transport.[26]

$$zT = \frac{S^2(\eta)}{\frac{(k_B/e)^2}{B \cdot F_0(\eta)} + L(\eta)}, \text{(S1)}$$

$$B = \left(\frac{k_B}{e}\right)^2 \cdot \frac{\sigma_{E_0} T}{\kappa_L} = \frac{2\hbar k_B^2}{3\pi} \cdot \frac{{v_l}^2 d N_v T}{m_I^* \Xi^2 \kappa_L}, \text{(S2)}$$

$$\sigma_{E_0} = \frac{2\hbar e^2}{3\pi} \cdot \frac{N_v v_l^2 d}{m_I^* \Xi^2}, \text{(S3)}$$

Where k_B is the Boltzmann constant, $\hbar$ is the reduced Planck constant, S is the Seebeck coefficient, η is the reduced Fermi energy, F_j is the Fermi integral, L is the Lorenz number, N_v is band degeneracy, v_l is the speed of sound, d is the sample density, and m_I^* is the inert effective mass, and σ_{E_0} is the transport coefficient.

Supplementary Note 3: The derivation of unique quality factor

For n-type $Pb_7Bi_4Se_{13}$, the electron is the major carrier. We use the two-band model to derive this unique quality factor.

Firstly, several physical parameters, such as reduced Fermi level for conduction, valence bands and reduced band gap, are introduced as follows.

$$\eta_e = \frac{E_f}{k_B T}, \text{(S4)}$$

$$\xi = \frac{E_g}{k_B T}, \text{(S5)}$$

$$\eta_h = -\eta_e - \xi = -\frac{E_f}{k_B T} - \frac{E_g}{k_B T}, \text{(S6)}$$

For Kane band, the Seebeck coefficient is shown as below.[25]

$$S = \frac{k_B}{e}\left(\frac{{}^1F_{-2}^1}{{}^0F_{-2}^1} - \eta\right), \text{(S7)}$$

And among them, the generalized Fermi integral is presented in this form:

$${}^nF_k^m(\eta) = \int_0^\infty \left(-\frac{\partial f}{\partial \varepsilon}\right)\varepsilon^n(\varepsilon + \alpha\varepsilon^2)^m[(1+2\alpha\varepsilon)^2+2]^{k/2}d\varepsilon, \text{(S8)}$$

in which, we have $\alpha = \frac{k_B T}{E_g}$ and total Seebeck coefficient, $S = \frac{S_e\sigma_e + S_h\sigma_h}{\sigma_e + \sigma_h}$ (S_e and S_h using different sign). Electrical conductivity ratio, $\gamma = \frac{\sigma_e}{\sigma_h} = \Lambda\frac{{}^0F_{-2}^1(\eta_e)}{{}^0F_{-2}^1(\eta_h)}$, and Λ is electronic band asymmetry, $\Lambda = \lambda \cdot \frac{N_{v,e}}{m_{I,e}^*\Xi_e^2} / \frac{N_{v,h}}{m_{I,h}^*\Xi_h^2}$. This Λ demonstrates the difference between conduction and valence bands with considering band degeneracy N_v, inertial effective mass m_I^* and deformation potential Ξ. It should be noted that Λ somewhat deviates away from the value based on acoustic phonon scattering (APS) dominated charge transport, especially considering the complex scattering mechanism existed in compounds, such as intervalley scattering, ionized impurity scattering, alloying scattering, *etc.* The parameter λ is a prefactor. The subscripts, *e* and *h,* denote electron and hole.[27]

To consider the band nonparabolicity and bipolar effect, we take both conduction band and valence band into consideration, as well as the bipolar thermal conductivity, k_b. The zT is expressed as follows:

$$
\begin{aligned}
zT &= \frac{S^2\sigma T}{\kappa_L+\kappa_c+\kappa_b} \\
&= \frac{S^2}{\frac{\kappa_L}{\sigma T}+\frac{\kappa_c}{\sigma T}+\frac{\kappa_b}{\sigma T}}
\end{aligned}, \text{(S9)}
$$

I. For the first term in the denominator, $\frac{\kappa_L}{\sigma T}$, it is rewritten as follows.

$$
\begin{aligned}
\frac{\kappa_L}{\sigma T} &= \frac{\kappa_L}{(\sigma_e+\sigma_h)T} = \frac{\kappa_L}{\sigma_e\left(1+\frac{1}{\gamma}\right)T} = \frac{\gamma}{\gamma+1}\cdot\frac{\kappa_L}{\sigma_e T} \\
&= \frac{\gamma}{\gamma+1}\cdot\frac{\left(k_B/e\right)^2}{\left(k_B/e\right)^2\cdot\frac{3\sigma_{E_0}\cdot {}^0F_{-2}^1}{\kappa_L}T} \\
&= \frac{\gamma}{\gamma+1}\cdot\frac{\left(k_B/e\right)^2}{\left(k_B/e\right)^2\cdot\frac{3\sigma_{E_0}\cdot {}^0F_{-2}^1}{\kappa_L}\cdot\frac{E_g}{k_B\xi}} \\
&= \frac{\gamma}{\gamma+1}\cdot\frac{\left(k_B/e\right)^2}{\left(k_B/e\right)^2\cdot\frac{3\sigma_{E_0}}{\kappa_L}\cdot\frac{E_g}{k_B}\cdot {}^0F_{-2}^1}\cdot\xi \\
&= \frac{\gamma}{\gamma+1}\cdot\frac{\left(k_B/e\right)^2}{3B_{Kane}^*\cdot {}^0F_{-2}^1}\cdot\xi
\end{aligned}, \text{(S10)}
$$

This first term could be expressed as $\frac{\kappa_L}{\sigma T}=\frac{\gamma}{\gamma+1}\cdot\frac{\left(k_B/e\right)^2}{3B_{Kane}^*\cdot {}^0F_{-2}^1}\cdot\xi$. And the unique quality factor is derived as $B_{Kane}^*=\left(\frac{k_B}{e}\right)^2\cdot\frac{\sigma_{E_0}}{\kappa_L}\cdot\frac{E_g}{k_B}=\frac{k_B}{e^2}\cdot\frac{\sigma_{E_0}E_g}{\kappa_L}$, among which σ_{E_0} is the transport coefficient, $\sigma_{E_0}=\frac{\sigma_e}{3\cdot {}^0F_{-2}^1}$.

II. For the second term in the denominator $\frac{k_c}{\sigma T}$,

$$
\frac{k_c}{\sigma T}=L=\frac{L_e\sigma_e+L_h\sigma_h}{\sigma_e+\sigma_h}=\frac{L_e\gamma+L_h}{\gamma+1}, \text{(S11)}
$$

III. For the final term $\frac{\kappa_b}{\sigma T}$, it could be described as below.

$$
\begin{aligned}
\frac{\kappa_b}{\sigma T} &= \frac{1}{\sigma T}\cdot\frac{\sigma_e\sigma_h}{(\sigma_e+\sigma_h)}(S_e-S_h)^2T \\
&= \frac{\frac{\sigma_e}{\sigma_h}}{\left(\frac{\sigma_e}{\sigma_h}+1\right)^2}(S_e-S_h)^2 \\
&= \frac{\gamma}{(\gamma+1)^2}(S_e-S_h)^2
\end{aligned}, \text{(S12)}
$$

By combining the above equations, the zT could be written as follows

$$
\begin{aligned}
zT &= \frac{S^2\sigma T}{\kappa_L+\kappa_c+\kappa_b} \\
&= \frac{S^2}{\frac{\kappa_L}{\sigma T}+\frac{\kappa_c}{\sigma T}+\frac{\kappa_b}{\sigma T}} \\
&= \frac{\left(\frac{S_e\gamma+S_h}{\gamma+1}\right)^2}{\frac{\gamma}{\gamma+1}\cdot\frac{\left(k_B/e\right)^2}{3B^*_{Kane}\cdot{}^0F^1_{-2}}\cdot\xi+\frac{L_e\gamma+L_h}{\gamma+1}+\frac{\gamma}{(\gamma+1)^2}(S_e-S_h)^2} \\
&= \frac{(S_e\gamma+S_h)^2}{\gamma(\gamma+1)\cdot\frac{\left(k_B/e\right)^2}{3B^*_{Kane}\cdot{}^0F^1_{-2}}\cdot\xi+(\gamma+1)(L_e\gamma+L_h)+\gamma(S_e-S_h)^2} \\
&= \frac{(S_e\gamma+S_h)^2}{(\gamma+1)\left[\frac{\left(k_B/e\right)^2\cdot\gamma\xi}{3B^*_{Kane}\cdot{}^0F^1_{-2}}+(L_e\gamma+L_h)\right]+\gamma(S_e-S_h)^2}
\end{aligned}
, \text{(S13)}
$$

And finally, zT based on two-band Kane model could be expressed as the following equation.

$$
zT=\frac{(S_e\gamma+S_h)^2}{(\gamma+1)\left[\frac{\left(k_B/e\right)^2\cdot\gamma\xi}{3B^*_{Kane}\cdot{}^0F^1_{-2}}+(L_e\gamma+L_h)\right]+\gamma(S_e-S_h)^2}, \text{(S14)}
$$

Supplementary Note 4: Two Kane band model for a weak bipolar effect

Since there exists a weak bipolar effect, these contributions of major and minor carriers to thermoelectric parameters, including Seebeck coefficient, electrical conductivity and thermal conductivity are intertwined. It is necessary to decouple the major carrier contribution from that of the minor carrier by using a well-developed work by *Pan*, *et al.*[21]

$$n = n_e + n_p \text{, (S15)}$$

$$S = \frac{S_e \sigma_e + S_p \sigma_p}{\sigma_e + \sigma_p} \text{, (S16)}$$

$$\sigma = \sigma_e + \sigma_p \text{, (S17)}$$

$$\kappa = \kappa_L + k_c + k_b \text{, (S18)}$$

$$L = \frac{L_e \sigma_e + L_p \sigma_p}{\sigma_e + \sigma_p} \text{, (S19)}$$

$$n_H = \frac{(2m_d^* k_B T)^{3/2}}{3\pi^2 \hbar^3} \cdot \frac{[(2K+1) \cdot {}^0F_{-2}^{1}]^2}{3K(K+2) \cdot {}^0F_{-4}^{1/2}} \text{, (S20)}$$

$$\mu_H = \frac{e}{\sqrt{2} m_I^*} \cdot \frac{\pi \hbar^4 v_l^2 d}{\Xi^2 (m_b^* k_B T)^{3/2}} \cdot \frac{3K(K+2) \cdot 3^0F_{-4}^{1/2}}{(2K+1)^2 \cdot {}^0F_{-2}^{1}} \text{, (S21)}$$

$$\sigma = n_H e \mu_H = \frac{2\hbar e^2}{\pi} \cdot \frac{N_v v_l^2 d}{m_I^* \Xi^2} \cdot {}^0F_{-2}^{1} \text{, (S22)}$$

$$L = \left(\frac{k_B}{e}\right)^2 \left[\frac{{}^2F_{-2}^{1}}{{}^0F_{-2}^{1}} - \left(\frac{{}^1F_{-2}^{1}}{{}^0F_{-2}^{1}}\right)^2\right] \text{, (S23)}$$

The above expressions include a series of fundamental parameters. The critical parameters are effective mass, band degeneracy, mobility and deformational potential, which could be summarized into a significant parameter, weighted mobility, μ_W.

$$\mu_W = \mu_0 \left(\frac{m_s^*}{m_e}\right)^{3/2} \text{, (S24)}$$

In the $Pb_7Bi_4Se_{13}$ based compounds, the electron is the major carrier and the hole is the minor carrier. The chemical potential of electron could be defined by $\eta_e = \frac{E_f}{k_B T}$.

And the chemical potential of hole is denoted as $\eta_p = -\eta_e - \frac{E_g}{k_B T}$. All charge transport parameters could be obtained after the determination of chemical potential and weighted mobilities. At first, the charge conduction at room temperature could be ascribed to the electron due to the enough band gap and almost unchanged carrier concentration shown in the inset of Fig. 3c of the main text. Based on this, the chemical potential and electron weighted mobility could be obtained. Secondly, by assuming the temperature dependence of chemical potential and weighted mobility, the remaining charge transport properties at higher temperatures could be obtained. It should be noted that the temperature dependence exponent could be changed slightly due to the existing diverse crystal defects, which introduce multiple charge scattering mechanisms. The temperature dependent parameters are tabulated in Table S3.

TableS3. Physical parameters for two-band modeling

T (K)	η_e	$\mu_{w,e}$ ($cm^2V^{-1}s^{-1}$)	$\mu_{w,p}$ ($cm^2V^{-1}s^{-1}$)	σ_e (Scm^{-1})	σ_p (Scm^{-1})	L_e ($10^{-8}W\Omega K^{-2}$)	L_p ($10^{-8}W\Omega K^{-2}$)	κ_c ($Wm^{-1}K^{-1}$)
**300**	3.2	24	400	234	0	2.01	1.49	0.14
**350**	2.4	23	322	218	0	1.91	1.49	0.15
**400**	1.8	22	267	198	0	1.83	1.49	0.14
**450**	1.3	21	227	178	0	1.76	1.49	0.14
**500**	0.8	21	196	159	0	1.70	1.49	0.14
**550**	0.5	20	171	144	0	1.66	1.49	0.13
**600**	0.2	19	152	132	0	1.63	1.49	0.13
**650**	0.0	19	136	123	1	1.61	1.49	0.13
**700**	-0.2	19	122	117	1	1.59	1.49	0.13
**750**	-0.4	18	111	113	2	1.58	1.49	0.14
**800**	-0.5	18	101	110	3	1.57	1.49	0.14

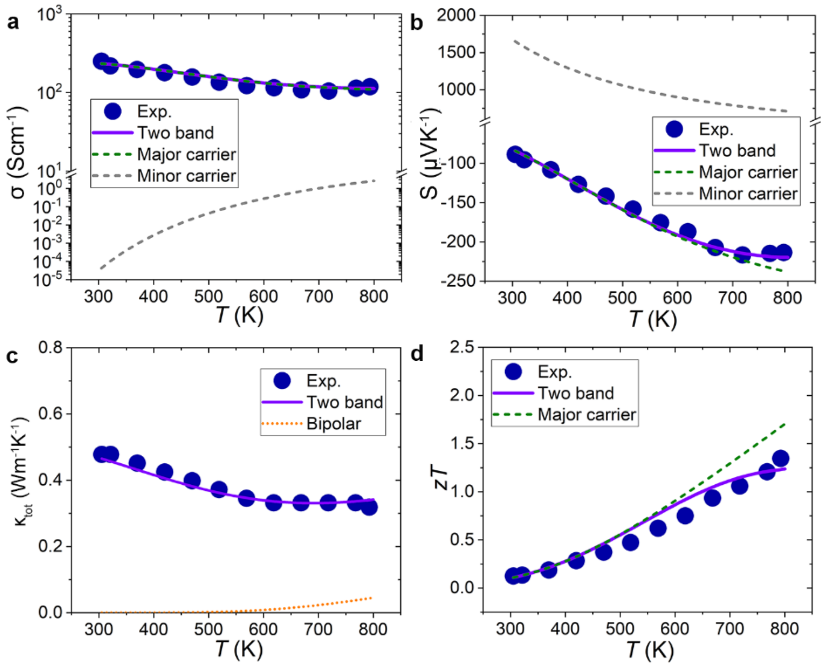

Fig. S11. Two-band modeling for **a** electrical conductivity, **b** Seebeck coefficient, **c** thermal conductivity, and **d** *zT* value. The symbol denotes the experimental data. The solid purple line demonstrates the data of two-band modeling. And the olive and gray dash lines represent contributions from major and minor carriers.

Here by using the two-band model demonstrated in the Section 4, the physical parameters could be decoupled from major and minor carriers. And the experimental data could be fitted reasonably as shown in Fig. S11. Considering the band gap, the contribution from the minor carrier is not so strong at high temperatures. The bipolar and carrier thermal conductivity are be estimated to be 0.05 Wm^{-1}K^{-1} and 0.14 Wm^{-1}K^{-1}. Consequently, the lattice thermal conductivity is estimated to be 0.14 Wm^{-1}K^{-1}, in a good agreement with the value estimated in the main text. These parameters obtained by this method are reliable and acceptable. It should be noted that the fit is not unique. Other sets of parameters might also offer reasonable simulations. For the calculation of quality factor B^*_{Kane}, the physical parameters of the major carrier are estimated from the two-band model and tabulated in TableS5.

TableS4. Physical parameters for the estimation of B^*_{Kane}

Sample	S_e (μVK^{-1})	η_e	σ_e (Scm^{-1})	κ_L (Wm^{-1}K^{-1})	ξ	B^*_{Kane}
Ga2	-238	-0.5	110	0.14	4.6	6

Supplementary Note 5: Different quality factors and applications

1. Quality factor by single parabolic band (SPB) model

$$zT = \frac{S^2 \sigma T}{\kappa_L + \kappa_e} = \frac{S^2}{\frac{\kappa_L}{\sigma T} + L} = \frac{S^2}{\frac{\left(k_B/e\right)^2}{\left(k_B/e\right)^2 \cdot \frac{\sigma_{E_0}}{\kappa_L} T \cdot F_0} + L} = \frac{S^2}{\frac{\left(k_B/e\right)^2}{B_{Para} \cdot F_0} + L}, \text{(S25)}$$

$$zT = \frac{S^2 \sigma T}{\kappa_L + \kappa_e} = \frac{S^2}{\frac{\left(k_B/e\right)^2}{B_{Para} \cdot F_0} + L}, \text{(S26)}$$

Quality factor, $B_{Para} = \left(\frac{k_B}{e}\right)^2 \frac{\sigma_{E_0}}{\kappa_L} T$, (S27)

Transport coefficient, $\sigma_{E_0} = \frac{\sigma}{F_0} = \frac{2\hbar e^2}{3\pi} \cdot \frac{N_v v_l^2 d}{m_I^* \Xi^2}$, (S28)

2. Quality factor by single Kane band (SKB) model

$$zT = \frac{S^2 \sigma T}{\kappa_L + \kappa_e} = \frac{S^2}{\frac{\kappa_L}{\sigma T} + L} = \frac{S^2}{\frac{\left(k_B/e\right)^2}{\left(k_B/e\right)^2 \cdot \frac{\sigma_{E_0}}{\kappa_L} T \cdot 3\,{}^0F_{-2}^1} + L} = \frac{S^2}{\frac{\left(k_B/e\right)^2}{B_{Kane} \cdot 3\,{}^0F_{-2}^1} + L}, \text{(S29)}$$

$$zT = \frac{S^2 \sigma T}{\kappa_L + \kappa_e} = \frac{S^2}{\frac{\left(k_B/e\right)^2}{B_{Kane} \cdot 3\,{}^0F_{-2}^1} + L}, \text{(S30)}$$

Quality factor, $B_{Kane} = \left(\frac{k_B}{e}\right)^2 \frac{\sigma_{E_0}}{\kappa_L} T$, (S31)

Transport coefficient, $\sigma_{E_0} = \frac{\sigma}{3 \cdot {}^0F_{-2}^1} = \frac{2\hbar e^2}{3\pi} \cdot \frac{N_v v_l^2 d}{m_I^* \Xi^2}$, (S32)

3. Quality factor by two band Kane band (TKB) model

$$
\begin{aligned}
zT &= \frac{S^2\sigma T}{\kappa_L+\kappa_c+\kappa_b} \\
&= \frac{S^2}{\frac{\kappa_L}{\sigma T}+\frac{\kappa_c}{\sigma T}+\frac{\kappa_b}{\sigma T}} \\
&= \frac{\left(\frac{S_e\gamma+S_h}{\gamma+1}\right)^2}{\frac{\gamma}{\gamma+1}\cdot\frac{\left(k_B/e\right)^2}{3B^*_{Kane}\cdot {}^0F^1_{-2}}\cdot\xi+\frac{L_e\gamma+L_h}{\gamma+1}+\frac{\gamma}{(\gamma+1)^2}(S_e-S_h)^2} \quad \text{, (S33)} \\
&= \frac{(S_e\gamma+S_h)^2}{\gamma(\gamma+1)\cdot\frac{\left(k_B/e\right)^2}{3B^*_{Kane}\cdot {}^0F^1_{-2}}\cdot\xi+(\gamma+1)(L_e\gamma+L_h)+\gamma(S_e-S_h)^2} \\
&= \frac{(S_e\gamma+S_h)^2}{(\gamma+1)\left[\frac{\left(k_B/e\right)^2\cdot\gamma\xi}{3B^*_{Kane}\cdot {}^0F^1_{-2}}+(L_e\gamma+L_h)\right]+\gamma(S_e-S_h)^2}
\end{aligned}
$$

$$zT = \frac{S^2\sigma T}{\kappa_L+\kappa_c+\kappa_b} = \frac{(S_e\gamma+S_h)^2}{(\gamma+1)\left[\frac{\left(k_B/e\right)^2\cdot\gamma\xi}{3B^*_{Kane}\cdot {}^0F^1_{-2}}+(L_e\gamma+L_h)\right]+\gamma(S_e-S_h)^2} \text{, (S34)}$$

Quality factor, $B^*_{Kane} = \frac{k_B}{e^2}\cdot\frac{\sigma_{E_0}E_g}{\kappa_L}$, (S35)

Transport coefficient, $\sigma_{E_0} = \frac{\sigma}{3\cdot {}^0F^1_{-2}} = \frac{2\hbar e^2}{3\pi}\cdot\frac{N_v v_l^2 d}{m_I^*\,\Xi^2}$, (S36)

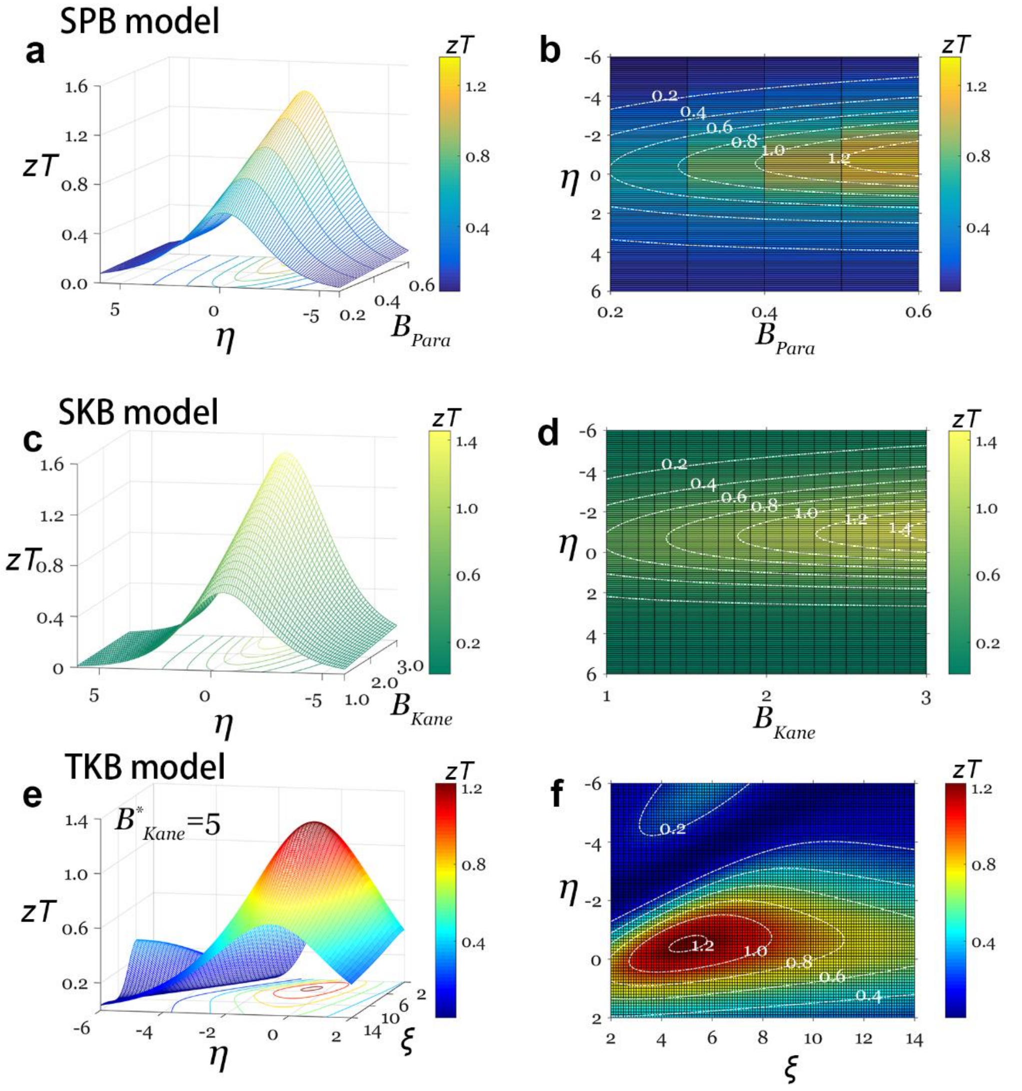

Fig. S12. a 3D plot of Figure of merit, *zT* as functions of reduced Fermi level, η and quality factor, B_{Para}. **b** Contour plot of η- and B_{Para}-dependent *zT*. The white dash lines show a series of *zT* values from 0.2 to 1.2. This B_{Para} is established on the single parabolic band model (SPB). **c** 3D plot of Figure of merit, *zT* as functions of reduced Fermi level, η and quality factor, B_{Kane}. **d** Contour plot of η- and B_{Kane}-dependent *zT*. The white dash lines show a series of *zT* values from 0.2 to 1.4. This B_{Kane} is established on the single Kane band model (SKB). **e** 3D plot of Figure of merit, *zT* as functions of reduced Fermi level, η and reduced band gap, ξ with quality factor, $B^{*}_{Kane} = 5$. **f** Contour plot of η- and ξ-dependent *zT*. The white dash lines show a series of *zT* values from 0.2 to 1.2. This B_{Kane} is established on the two Kane band model (TKB).

Fig.S12 shows different quality factors established on SPB, SKB, and TKB models, respectively. The quality factor with SPB model is widely used in thermoelectric materials with wide band gaps, such as PbSe, SnSe, BiCuSeO, *et al*. For thermoelectric materials with narrow bandgaps, the band nonparabolicity is inevitable throughout the entire temperature ranges. The quality factor with single Kane band

model is utilized at room temperature or moderate temperature, such as La- and I-doped PbTe. Untill now, there is no report on quality factor both considering band nonparabolity and bipolar effect, which greatly limit the precise evaluation and prediction of thermoelectric materials with narrow band gaps, such as PbTe, Bi_2Se_3, $CoSb_3$, SnTe, *et al* and unconventional Pb_7Bi4Se_{13} here. Here, the unique quality factor, B^*_{Kane}, established on the TKB model, considers the band nonparabolicity and bipolar effect, which leads to more accurate and reliable results.

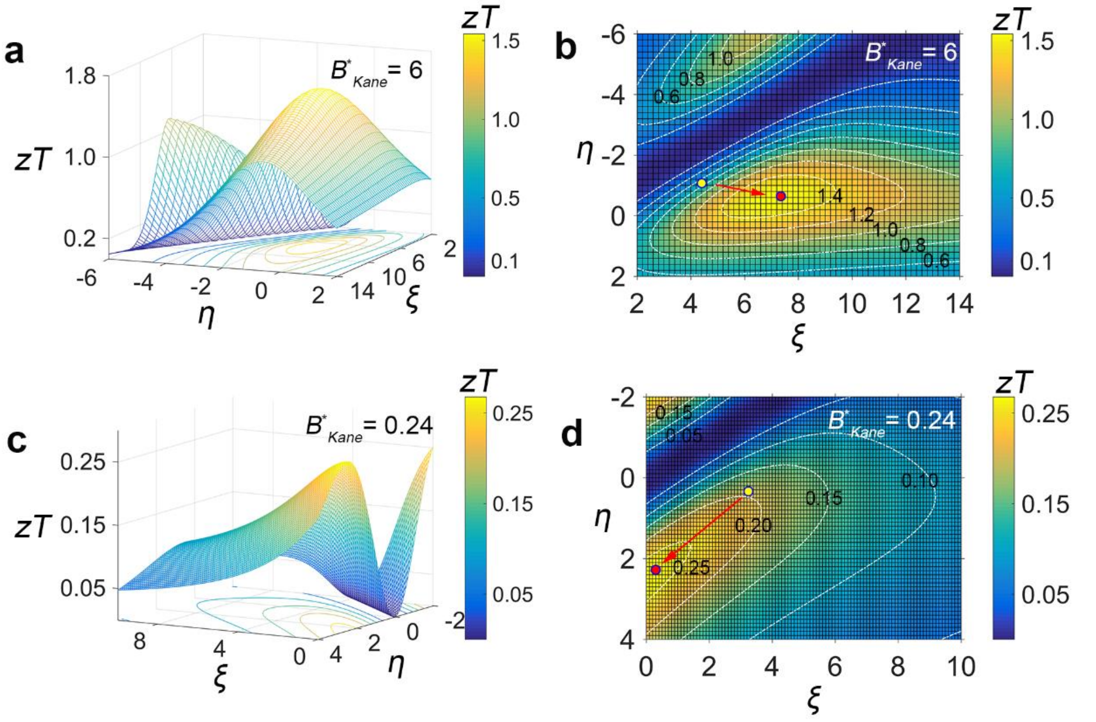

Fig. S13. a 3D plot of Figure of merit, *zT* as functions of reduced Fermi level, η and reduced band gap, ξ with quality factor, B^*_{Kane} = 6. **b** Contour plot of η- and ξ-dependent *zT*. The white dash lines show a series of *zT* values from 0.6 to 1.4. The white point comes from the experiment data of $(Bi,Sb)_2Se_3$. The red point is the predicted highest *zT* value. **c** 3D plot of Figure of merit, *zT* as functions of reduced Fermi level, η and reduced band gap, ξ with quality factor, B^*_{Kane} = 0.24. **d** Contour plot of η- and ξ-dependent *zT*. The white dash lines show a series of *zT* values from 0.10 to 0.25. This B^*_{Kane} is established on the two Kane band model (TKB).

This unique quality factor could work well in $Pb_7Bi_4Se_{13}$ as discussed in the main text. Here we will extend its application in different thermoelectric materials with narrow gaps. To extend the application of this unique quality factor, we firstly apply it into the typical thermoelectric material, $(Bi,Sb)_2Te_3$ with a narrow band gap of ~ 0.18 eV. Fig. S13a-b show the 3D plot and contour plot of Figure of merit, *zT* as functions of reduced Fermi level, η and reduced band gap, ξ in $(Bi,Sb)_2Te_3$. The unique quality factor, B^*_{Kane} is calculated to be 6 by using physical parameters from *Pan et al*.[21] The quality factor derivation has been described in the section 4. In Fig. S13b, the experiment data is revealed by the white point with the measured *zT* of ~ 0.76. The highest *zT* is predicted to be 1.54, shown by the red point. The red arrow indicates the optimization of η and ξ to achieve the highest *zT*. Not only can the unique quality factor

be applied into the typical thermoelectric material, it also can be adopted into the recently reported van der Waals crystal Ta_4SiTe_4, which exhibits a narrow band gap of ~ 0.08eV.[28] At low temperature, such as 50 K, it is reasonable to assume there is only one kind of carrier in Ta_4SiTe_4. Consequently, the weighted mobility of electron and chemical potential can be derived. By assuming the linear temperature dependence of chemical potential, the remaining parameters could be obtained. The B^*_{Kane} is calculated to be 0.24 for Ta_4SiTe_4 at 300 K as shown in Fig. S13c. The experiment zT is 0.18, which could be optimized to the highest zT of 0.27 shown by the red arrow.

The following table shows the physical parameters to derivate the B^*_{Kane}.

Table S5. Physical parameters for the estimation of B^*_{Kane} in typical $(Bi,Sb)_2Te_3$ and van der Waals crystal $Ta_4(Si,P)Te_4$

Sample	S (μVK^{-1})	η	σ (Scm^{-1})	κ_L (Wm^{-1}K^{-1})	ξ	B^*_{Kane}
$Bi_{0.4}Sb_{1.6}Te_3$	268	-0.9	402	0.31	4.4	6
$Ta_4Si_{0.995}P_{0.005}Te_4$	168	0.7	216	0.82	3.1	0.24

Supplementary Note 6: Thermal conductivity investigation

Table S6. The speed of sound and related physical parameters of representative thermoelectric materials with low thermal conductivities

**Compound**	v_L (ms^{-1})	v_T (ms^{-1})	v_m (ms^{-1})	γ_G	$\boldsymbol{\Theta_D}$ **(K)**	**Literature**
$AgSbTe_2$	3123	1538	1727	2.1	125	29
Cu_7PSe_6	3580	1870	2092	1.9	215	30
$K_2Bi_8Se_{13}$	2683	1438	1605	1.8	154	31
Ag_9GaSe_6	2916	1222	1382	2.6	147	32
BiSe	2950	1618	1804	1.7	319	23
$Cu_{17.6}Fe_{17.6}S_{32}$	3039	1842	2036	1.6	230	33
**$\mathbf{Pb_7Bi_4Se_{13}}$**	**2842**	**1350**	**1553**	**2.2**	**148**	**This work**

Debye-Callaway model

$$\kappa_L = \frac{k_B}{2\pi^2 v}\left(\frac{k_B T}{\hbar}\right)^3 \int_0^{\Theta_D/T} \frac{\tau(x) x^4 e^x}{(e^x - 1)^2} dx, \text{ (S37)}$$ [34]

where $x = \frac{\hbar\omega}{k_B T}$, Θ_D is the Debye temperature and $\tau(x)$ is the total phonon relaxation time. This $\tau(x)$ can be written as $\tau^{-1} = \tau_U^{-1} + \tau_{PD}^{-1} + \tau_B^{-1} + \tau_{DS}^{-1} + \tau_{NP}^{-1}$, which includes Normal and Umklapp processes of phonon-phonon (N and U) scattering, point defect (PD), ground boundary (B), dislocation (DS) and nano-scale precipitate (NP) scattering.

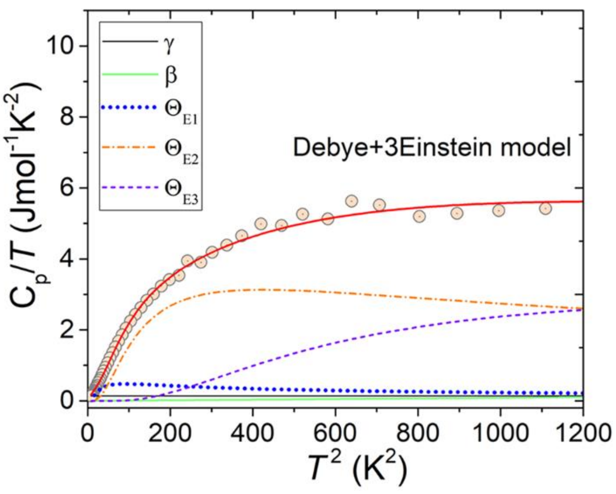

Fig. S13. C_p/T as a function of T^2 by using the Debye and three Einstein model.

Table S7. Parameters on the Debye-Einstein model on C_p measurement

Parameter	Value
γ' (10^{-1} Jmol^{-1}K^{-2})	1.204
β (10^{-5} J mol^{-1}K^{-4})	8.455
Θ_D (K)†	148
Θ_{E1} (K)	13 (9 cm^{-1})
Θ_{E2} (K)	33 (23 cm^{-1})
Θ_{E3} (K)	119 (83 cm^{-1})

† This Debye temperature is acquired by fitting the C_p versus T^2, also consistent with the Debye temperature from speed of sound measurement.

$$\frac{C_p}{T} = \gamma' + \beta T^2 + \sum_i \left(A_i (\Theta_{Ei})^2 \cdot (T^2)^{-3/2} \cdot \frac{e^{\Theta_{Ei}/T}}{(e^{\Theta_{Ei}/T} - 1)^2} \right), \text{(S38)}$$

In this model, the first term γ' denotes the Sommerfeld constant and represents the electronic contribution. And the second term stands for the Debye lattice component with $\beta = B\left(\frac{12\pi^4 N_A k_B}{5}\right)\Theta_D^{-3}$, where N_A is Avogadro's number, k_B is the Boltzmann constant, and Θ_D is the Debye temperature. The parameter B is revealed as $B = 1 - \sum_i \frac{A_i}{3NR}$, where N is the number of atoms per formula unit and R is the gas constant (8.314 Jmol^{-1}K^{-1}). The third summation term highlights the localized Einstein oscillators, in which A_i is the prefactor of each Einstein mode.

Supplementary Note 7: Full phonon spectrum and phonon density of states

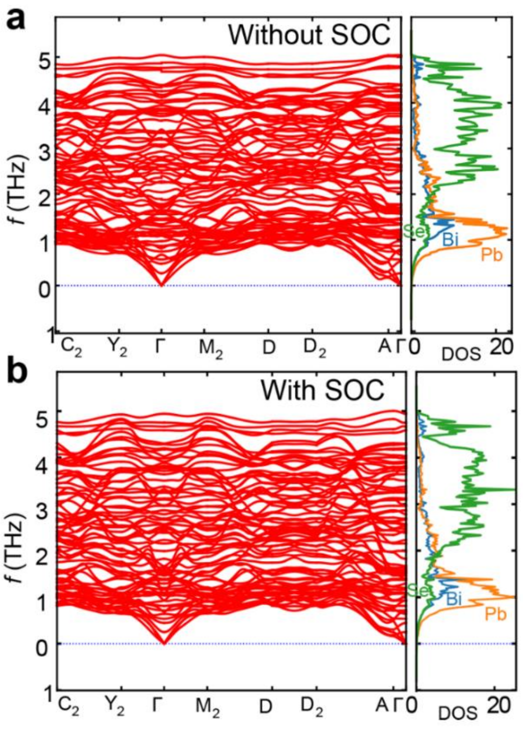

Fig. S14. Calculated phonon dispersions and phonon density of states (DOS) of $Pb_7Bi_4Se_{13}$ **a** without SOC and **b** with SOC.

To investigate the SOC effect on phonon dispersion, we also performed the phonon calculations without and with SOC. Fig. S14 shows the phonon dispersions and phonon density of states (DOS) calculated from the finite-displacement and supercell method (48 supercells in each calculation). We can find that the SOC effect has a slight effect on the overall dispersions. The Γ-point phonons in both calculations show the largest frequency of around 5 THz. In particular, the SOC effect on the low-frequency phonon dispersions are insignificant. Therefore, the effect of SOC on phonon transport of $Pb_7Bi_4Se_{13}$ should be limited, considering that the low thermal conductivity is mainly dominated by the low-frequency phonons.

Combining the appended electronic band calculations including SOC with the phonon calculations including SOC, we can conclude that the SOC should have small effect on the thermoelectric performance of $Pb_7Bi_4Se_{13}$.

Supplementary Note 8: Micro-structural investigation

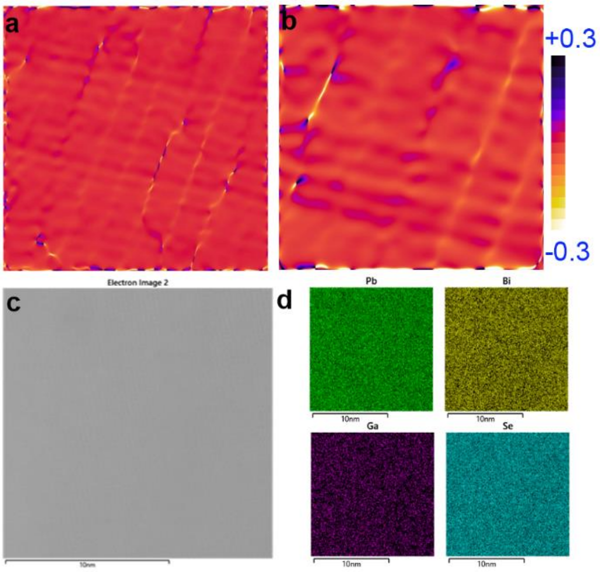

Fig. S15. a and **b** GPA results along *yy* direction, corresponding to the regions shown by Fig. 6a and 6b of the main text, respectively. The scale bar is also shown. **c** STEM image. **d** Corresponding EDS mapping for Pb, Bi, Ga and Se. The elemental analysis shows that the homogeneously distributed elements in the periodic lattice defects after investigating many regions.

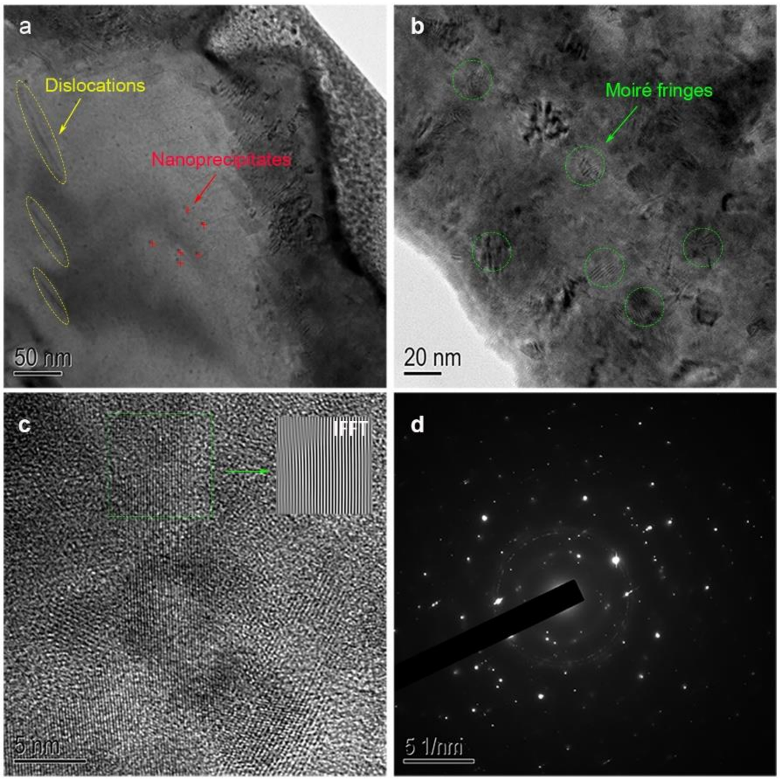

Fig. S16. a Low-magnification TEM image with a high number density of nanoprecipitates marked by the red crosses, and dislocations demonstrated by the yellow ellipses. **b** Low-magnification TEM image with numerous moiré fringes distributed in the host matrix. **c** High-resolution TEM image with nanoprecipitates and the corresponding inverse fast Fourier transform (IFFT). **d** Selected area electron diffraction (SAED).

References

1. Ohta M, Chung DY, Kunii M, Kanatzidis MG. Low lattice thermal conductivity in Pb5Bi6Se14, Pb3Bi2S6, and PbBi2S4: promising thermoelectric materials in the cannizzarite, lillianite, and galenobismuthite homologous series. *Journal of Materials Chemistry A* **2**, 20048-20058 (2014).

2. Heinke F, Nietschke F, Fraunhofer C, Dovgaliuk I, Schiller J, Oeckler O. Structure and thermoelectric properties of the silver lead bismuth selenides Ag5Pb9Bi19Se40 and AgPb3Bi7Se14. *Dalton Transactions* **47**, 12431-12438 (2018).

3. Casamento J*, et al.* Crystal structure and thermoelectric properties of the7,7L Lillianite homologue Pb6Bi2Se9. *Inorg Chem* **56**, 261-268 (2017).

4. Li J*, et al.* Thermoelectric Material SnPb2Bi2S6: The 4, 4L Member of Lillianite Homologous Series with Low Lattice Thermal Conductivity. *Inorg Chem* **58**, 1339-1348 (2018).

5. Sassi S*, et al.* Crystal Structure and Transport Properties of the Homologous Compounds (PbSe) 5(Bi2Se3)3m (m= 2, 3). *Inorg Chem* **57**, 422-434 (2018).

6. Lu R*, et al.* High carrier mobility and ultralow thermal conductivity in the synthetic layered superlattice Sn4Bi10Se19. *Materials Advances*, (2021).

7. Heinke F*, et al.* Cornucopia of Structures in the Pseudobinary System (SnSe)xBi2Se3: A Crystal-Chemical Copycat. *Inorg Chem* **57**, 4427-4440 (2018).

8. Chen K-B, Lee C-S. Synthesis and characterization of quaternary selenides Sn2Pb5Bi4Se13 and Sn8. 65Pb0. 35Bi4Se15. *Solid state sciences* **11**, 1666-1672 (2009).

9. Mrotzek A, Kanatzidis MG. Tropochemical Cell-Twinning in the New Quaternary Bismuth Selenides K x Sn6-2xBi2+xSe9 and KSn5Bi5Se13. *Inorg Chem* **42**, 7200-7206 (2003).

10. Agha EC*, et al.* LiPbSb3S6: A semiconducting sulfosalt with very low thermal conductivity. *Inorg Chem* **53**, 673-675 (2014).

11. Shelimova L, Karpinskii O, Konstantinov P, Avilov E, Kretova M, Zemskov V. Crystal Structures and Thermoelectric Properties of Layered Compounds in the ATe–Bi2Te3 (A= Ge, Sn, Pb) Systems. *Inorganic Materials* **40**, 451-460 (2004).

12. Shelimova L*, et al.* Anisotropic thermoelectric properties of the layered compounds PbSb2Te4 and PbBi4Te7. *Inorganic Materials* **43**, 125-131 (2007).

13. Zhao J*, et al.* Semiconducting pavonites CdMBi4Se8 (M= Sn and Pb) and their thermoelectric

properties. *Chem Mater* **29**, 8494-8503 (2017).

14. Hwang J-Y, Ahn JY, Lee KH, Kim SW. Structural optimization for thermoelectric properties in Cu-Bi-S pavonite compounds. *Journal of Alloys and Compounds* **704**, 282-288 (2017).

15. Zhao J*, et al.* Six quaternary chalcogenides of the pavonite homologous series with ultralow lattice thermal conductivity. *Chem Mater* **31**, 3430-3439 (2019).

16. Khoury JF*, et al.* Quaternary Pavonites A1+xSn2–xBi5+xS10 (A+= Li+, Na+): Site Occupancy Disorder Defines Electronic Structure. *Inorg Chem* **57**, 2260-2268 (2018).

17. Sassi S*, et al.* Thermoelectric Properties of Polycrystalline n-Type Pb5Bi6Se14. *Journal of Electronic Materials* **46**, 2790-2796 (2017).

18. Sotnikov AV, Jood P, Ohta M. Enhancing the Thermoelectric Properties of Misfit Layered Sulfides (MS)1.2+q(NbS2)n (M= Gd and Dy) through Structural Evolution and Compositional Tuning. *ACS omega* **5**, 13006-13013 (2020).

19. Djieutedjeu H*, et al.* Crystal Structure, Charge Transport, and Magnetic Properties of MnSb2Se4.). Wiley Online Library (2011).

20. Dawahre L*, et al.* Lone-Electron-Pair Micelles Strengthen Bond Anharmonicity in MnPb16Sb14S38 Complex Sulfosalt Leading to Ultralow Thermal Conductivity. *ACS Applied Materials & Interfaces* **12**, 44991-44997 (2020).

21. Pan Y*, et al.* Synergistic modulation of mobility and thermal conductivity in (Bi, Sb)2Te3 towards high thermoelectric performance. *Energ Environ Sci* **12**, 624-630 (2019).

22. Deng RG*, et al.* Thermal conductivity in Bi0.5Sb1.5Te3+x and the role of dense dislocation arrays at grain boundaries. *Sci Adv* **4**, (2018).

23. Samanta M, Pal K, Pal P, Waghmare UV, Biswas K. Localized Vibrations of Bi Bilayer Leading to Ultralow Lattice Thermal Conductivity and High Thermoelectric Performance in Weak Topological Insulator n-Type BiSe. *J Am Chem Soc* **140**, 5866-5872 (2018).

24. LaLonde AD, Pei Y, Snyder GJ. Reevaluation of PbTe1− xIx as high performance n-type thermoelectric material. *Energ Environ Sci* **4**, 2090-2096 (2011).

25. Pei YZ, LaLonde AD, Wang H, Snyder GJ. Low effective mass leading to high thermoelectric performance. *Energ Environ Sci* **5**, 7963-7969 (2012).

26. Kang SD, Snyder GJ. Transport Property Analysis Method for Thermoelectric Materials: Material Quality Factor and the Effective Mass Model. *arXiv:physics* **1710.06896**, (2017).

27. Zhu HT, *et al.* Understanding the asymmetrical thermoelectric performance for discovering promising thermoelectric materials. *Sci Adv* **5**, (2019).

28. Xu Q, *et al.* Thermoelectric properties of phosphorus-doped van der Waals crystal Ta4SiTe4. *Mater Today Phys*, 100417 (2021).

29. Ma J, *et al.* Glass-like phonon scattering from a spontaneous nanostructure in AgSbTe2. *Nat Nanotechnol* **8**, 445-451 (2013).

30. Weldert KS, Zeier WG, Day TW, Panthöfer M, Snyder GJ, Tremel W. Thermoelectric transport in Cu7PSe6 with high copper ionic mobility. *J Am Chem Soc* **136**, 12035-12040 (2014).

31. Pei YL, *et al.* Multiple Converged Conduction Bands in K2Bi8Se13: A Promising Thermoelectric Material with Extremely Low Thermal Conductivity. *J Am Chem Soc* **138**, 16364-16371 (2016).

32. Lin SQ, *et al.* High Thermoelectric Performance of Ag9GaSe6 Enabled by Low Cutoff Frequency of Acoustic Phonons. *Joule* **1**, 816-830 (2017).

33. Xie HY, *et al.* Origin of Intrinsically Low Thermal Conductivity in Talnakhite $Cu_{17.6}Fe_{17.6}S_{32}$ Thermoelectric Material: Correlations between Lattice Dynamics and Thermal Transport. *J Am Chem Soc* **141**, 10905-10914 (2019).

34. Toberer ES, Zevalkink A, Snyder GJ. Phonon Engineering Through Crystal Chemistry. *J Mater Chem* **21**, 15843-15852 (2011).
